# Indigenous impacts on north Australian savanna fire regimes over the Holocene

**DOI:** 10.1038/s41598-021-02618-z

**Published:** 2021-11-30

**Authors:** Christopher M. Wurster, Cassandra Rowe, Costijn Zwart, Dirk Sachse, Vladimir Levchenko, Michael I. Bird

**Affiliations:** 1 ARC Centre of Excellence for Australian Biodiversity and Heritage, James Cook University, PO Box 6811, Cairns, QLD 4870 Australia; 2 College of Science and Engineering, James Cook University, PO Box 6811, Cairns, QLD 4870 Australia; 3 Helmholtz Centre Potsdam, GFZ German Research Centre for Geosciences, Telegrafenberg, 14473 Potsdam, Germany; 4 Australian Nuclear Science and Technology Organization, Locked Bag 2001, Kirrawee DC, NSW 2232 Australia

**Keywords:** Geochemistry, Palaeoclimate

## Abstract

Fire is an essential component of tropical savannas, driving key ecological feedbacks and functions. Indigenous manipulation of fire has been practiced for tens of millennia in Australian savannas, and there is a renewed interest in understanding the effects of anthropogenic burning on savanna systems. However, separating the impacts of natural and human fire regimes on millennial timescales remains difficult. Here we show using palynological and isotope geochemical proxy records from a rare permanent water body in Northern Australia that vegetation, climate, and fire dynamics were intimately linked over the early to mid-Holocene. As the El Niño/Southern Oscillation (ENSO) intensified during the late Holocene, a decoupling occurred between fire intensity and frequency, landscape vegetation, and the source of vegetation burnt. We infer from this decoupling, that indigenous fire management began or intensified at around 3 cal kyr BP, possibly as a response to ENSO related climate variability. Indigenous fire management reduced fire intensity and targeted understory tropical grasses, enabling woody thickening to continue in a drying climate.

The long-term response of fire to changing climate and human management in tropical savanna remains poorly understood despite the essential role fire plays in potentially maintaining bistable states and modulating biodiversity^1–4^. In Australia, indigenous fire use is widely acknowledged as having occurred since human arrival at least 50,000 years ago, yet the impact of anthropogenic fire is still debated^2,5,6^. Although it is generally agreed that indigenous fire management has long been practiced^2,7^, separating the anthropogenic effects on fire regime from natural variability remains challenging.

In the context of tropical northern Australian savannas, the impacts and causes of changing fire regimes remains enigmatic. For example, it is still debated whether indigenous fire practices played a role in megafaunal extinction, or replaced the ecological function of megafauna^2,7^. More recently, there has been a recognition that the replacement of indigenous forest management with European fire management has led to cascading detrimental ecological costs in tropical savannas in Australia and globally^8^. As a result, there is a renewed interest in understanding and re-implementing indigenous fire management in Australia for ecological and carbon sequestration benefits^6^. Understanding the impacts of fire on ecosystems requires an assessment of long-term changes in fire regime and how variability in fire regime over time is controlled by climate–environment–human interactions.

Here we present a multi-proxy archive of changing environmental conditions directly comparable to independent records of fire frequency, fire intensity, and vegetation burnt. Girraween lagoon, in northern Australia is ideally positioned to explore changing fire regimes in the tropical savannas that cover roughly a quarter of Australia (Fig. 1). The lagoon represents a rare permanent water feature in the Australian savanna region, with a sedimentary record containing multiple environmental and fire proxies. A record of precipitation/evaporation balance (indicative of the strength of the monsoon) is based on the hydrogen isotope composition of leaf wax *n*-alkanes (expressed as *δ*D values)^9^, a biomarker derived from higher terrestrial plants (*δ*D_wax_)^10,11^. Local vegetation is inferred from the pollen record^12^, focusing on grass relative to dryland tree pollen flux (expressed as *f*_Grass_) as a means to represent local tree cover in the catchment over time. Fire frequencies are inferred from charcoal particle counts^12^, whereas fire intensities are based on the geochemical measurement of pyrogenic carbon mass accumulation rate (measured as Stable Polycyclic Aromatic Hydrocarbon or SPAC), as the proportion of SPAC in charcoal increases with increasing fire intensity^13,14^. The carbon isotope composition of SPAC (*δ*^13^C_SPAC_ value) is indicative of vegetation burnt in tropical savannas^15^ as grasses utilize the C_4_ photosynthetic pathway (c. − 11 to − 15‰) with a distinct carbon isotope composition when compared with C_3_ woody vegetation (c. − 24 to − 35‰)^16^.

**Figure 1:**
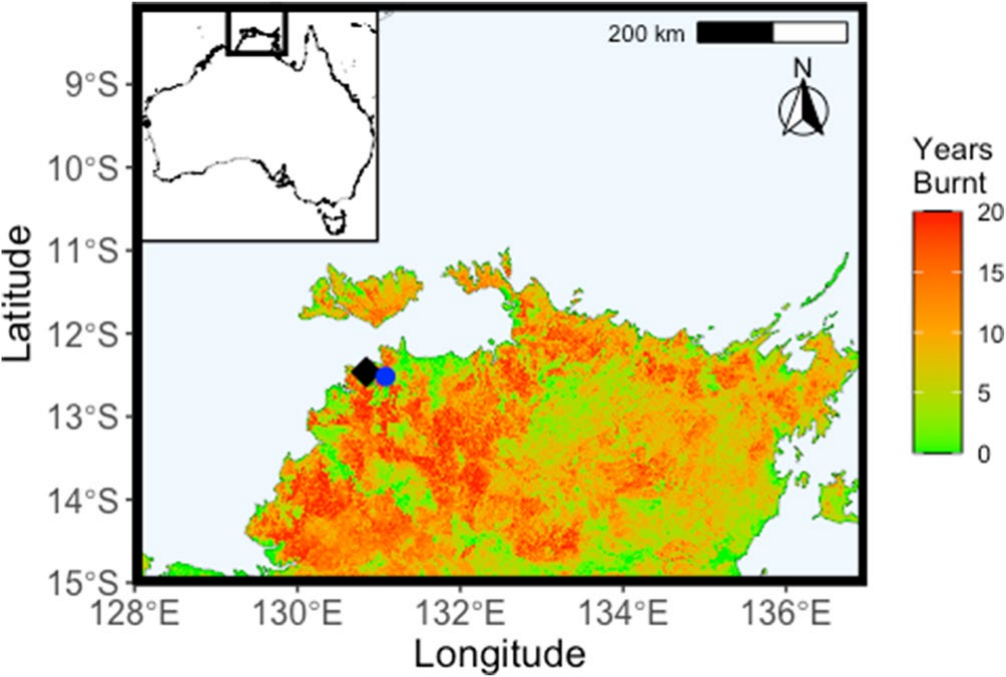
Map of study area showing location of Girraween Lagoon (blue circle) near to Darwin (black diamond). Figure created in in R (Version 4.0.0, https://www.r-project.org/) using regional fire frequency where ‘Years Burnt’ identifies the number of years a given polygon has been detected as being burnt over the 20-year period of 2000–2019^17^.

Our integrated multi-proxy results provide a unique opportunity to directly compare changes in environmental parameters, fire frequency, fire intensity, and vegetation burnt. In particular, our data show a strong coupling between monsoon intensity (*δ*D_wax_), vegetation (tree-grass ratio; *f*_Grass_), fire frequency, fire intensity, and vegetation burnt (*δ*^13^C_SPAC_) during the Holocene until ~ 3 cal kyr BP, after which fire regimes become decoupled from local vegetation and monsoon variability. We suggest that indigenous fire practices began or intensified at that time, possibly as a response to changing climate variability and/or population increases.

## Results

### The Early Holocene at Girraween: a dry grassland with intense fires

**Figure 2:**
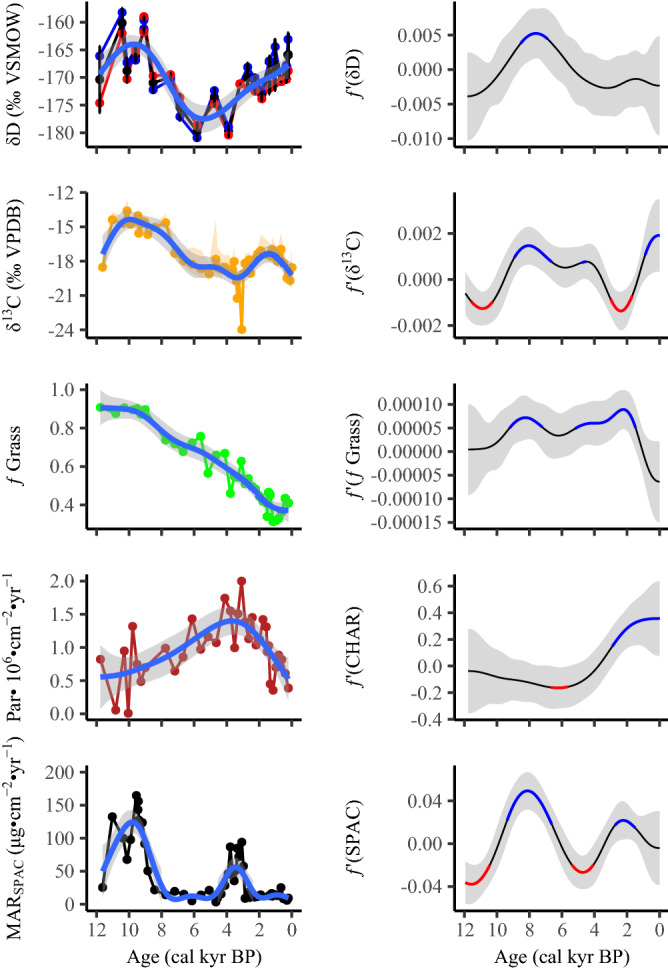
Multi-proxy diagram showing change in SPAC (stable polycyclic aromatic carbon) mass accumulation rate (as a proxy for fire intensity), charcoal particle count (as a proxy for fire frequency), the fraction of grass relative to total terrestrial pollen counts (as a proxy for vegetation), *δ*^13^C_SPAC_ values (as a proxy for source of burning), and *δ*D values of *n*-C_27_ and *n*-C_29_ alkanes (as a proxy for monsoon strength) from core GIR2 over the Holocene. The most recent sample for MAR_SPAC_ (326 μg cm^−2^ yr^−1^) is removed as it represents European induced fire regime. Also shown are Generalized additive models (GAMs) as a blue line through the GIR2 data with confidence levels (significance = 0.9) shaded. Corresponding right panels show first derivatives calculated from the GAM splines. Significant positive (negative) trends are highlighted in red (blue) with significance set to 0.975.

Although variable, the driest period in the Girraween record occurred at the start of the Holocene as evidenced by the highest *δ*D_wax_ values in the record which peaked between 10.5 and 9 cal kyr BP (Fig. 2). The vegetation surrounding the study site was dominated by tropical grasses with Poaceae representing > 75% of the total terrestrial pollen, peaking at ~ 90% between from at least the start of the Holocene until 9 cal kyr BP. Although fires may have been infrequent, as indicated by generally low charcoal particle flux, the highest MAR_SPAC_ in the record was measured between 11 and 9 cal kyr BP indicating that those fires which did occur were very intense. The *δ*^13^C_SPAC_ values range from − 15.7 to − 13.6‰ over this time, and indicate that almost all of the fuel load being burnt was derived from tropical grass (C_4_) biomass. Notably, Generalized Additive Models (GAMs) fitted to the proxy data indicate significant positive changes (proxy increases over time) early in the record occurred in MAR_SPAC_ and *δ*^13^C_SPAC_ values. When all proxies are considered together, the early Holocene appears to have been relatively dry and dominated by tropical grasses, with infrequent, yet intense fires that burnt the surrounding grassland.

### The mid-Holocene at Girraween: relatively wet with increasing arboreal vegetation that burned more frequently, but less severely

*δ*D_wax_ values began to decrease by 7.5 cal kyr BP, toward a broad minimum in the record between 6 and 3.8 cal kyr BP, indicating a trend to wetter conditions. Arboreal pollen becomes an increasingly important component, with tree pollen varying between 25 and 55% of terrestrial pollen counts. Charcoal particle flux also increased, suggesting fires became more frequent. Notably, there is an abrupt decrease in MAR_SPAC_ after ~ 9 cal kyr BP, and *δ*^13^C_SPAC_ values indicate that the biomass burnt had an increasingly woody (C_3_) character, decreasing from − 14.6 to approximately − 19.0‰. GAMs indicate that the mid-Holocene encompassed several significant periods of change including a period where *δ*D_wax_ values decreased significantly from 8.7 to ~ 6.4 cal kyr BP. Significant changes to lower *f*_Grass_ counts are noted between 9.2 to 7.2 and 5.2 to 1.4 cal kyr BP. Additionally, a significant change is evident in the amount of burnt woody vegetation from ~ 9 to 6.6 cal kyr BP, similar in timing to the lowest SPAC accumulation rates in the record. Moreover, a significant increase in charcoal particle flux is identified from ~ 6.6 to 5.6 cal kyr BP. Considering all proxy evidence together, the mid-Holocene was the wettest period in the record, with an increasingly woody character associated with an increased fire frequency, and an increased arboreal component in the biomass being burnt. However, fire intensity was low.

### The late Holocene: a return to drier conditions, but with a decoupling between fire records and vegetation

Although the *δ*D_wax_ values over this period indicate a change toward drier and variable conditions, similar the early Holocene, by 3 cal kyr BP, the *f*_Grass_ pollen continued to decrease to a minimum at ~ 1 cal kyr BP. Charcoal flux was highest at ~ 3 cal kyr BP, declining steadily afterwards. Most notably, there is a peak in fire intensity at ~ 3 cal kyr BP, contemporaneous with a notable and abrupt decrease in *δ*^13^C_SPAC_ values indicating that an increase in burnt woody biomass that was coincident with the abrupt increase in fire intensity. After this time, MAR_SPAC_ rate abruptly decreased to low values, and *δ*^13^C_SPAC_ values indicate increasing amounts of tropical grass biomass was being burnt. The GAMs identify significant changes in all proxy records just after the SPAC accumulation peak at ~ 3 cal kyr BP, with the exception of *δ*D_wax_ values, although late Holocene *δ*D_wax_ values increase distinctly.

While *δ*D_wax_ values were correlated significantly with every other proxy over the early to mid-Holocene, *δ*D_wax_ values do not correlate significantly with any other proxy over the late Holocene (Table 1). Early to mid-Holocene correlations with *δ*D_wax_ are all positive with the exception of charcoal particle flux, which was significantly but negatively correlated. Particularly strong early to mid-Holocene correlations are noted between *δ*D_wax_ and *δ*^13^C_SPAC_ (r = 0.74) and *f*_Grass_ (r = 0.78). Moreover, we note that charcoal particle flux is negatively correlated with other proxies, with the exception of MAR_SPAC_, which is not significantly correlated. The character of these correlations changes during the late Holocene, where no proxy is significantly related to *δ*D_wax_. Notably, several proxies that demonstrate significant relationships (*δ*^13^C_SPAC_ vs. MAR_SPAC_, *δ*^13^C_SPAC_ vs. the *f*_Grass_, and *f*_Grass_ vs. CHAR) show the opposite trends (slope) during the late Holocene compared with the early to mid-Holocene.

**Table 1:** Pearson’s correlation coefficients (r) for covariates.

Covariate	Covariate	Early to mid (> 3kyr BP)	Late (< 3 kyr BP)
*δ*D_wax_	δ^13^C_SPAC_	0.74, *P* = 0.006**	− 0.17, *P* = 0.687
	MAR_SPAC_	0.68, *P* = 0.015*	0.00, *P* = 1
	*f*_Grass_	0.71, *P* = 0.010**	− 0.26, *P* = 0.534
	CHAR	− 0.69, *P* = 0.013*	− 0.60, *P* = 0.116
*δ*^13^C_SPAC_	MAR_SPAC_	0.55, *P* = 0.002**	− 0.90, P < 0.001**
	*f*_Grass_	0.78, *P* = 0.009**	− 0.67, *P* = 0.002**
	CHAR	− 0.66, *P* = 0.002**	− 0.47, *P* = 0.049*
MAR_SPAC_	*f*_Grass_	0.42, *P* = 0.093	0.57, *P* = 0.011*
	CHAR	0.24, *P* = 0.322	0.49, *P* = 0.033*
*f*_Grass_	CHAR	− 0.62, *P* = 0.008**	0.68, *P* = 0.002**

**Indicates significance at 0.01, *indicates significance at 0.05. *δ*D_wax_ is the average *δ*D value of *n*-C_27_ and *n*-C_29_ *n*-alkane, *f*_Grass_ is the fraction of terrestrial grass that is Poaceae, CHAR is the charcoal particle flux, and MAR_SPAC_ indicates Stable Polyclyclic Aromatic Carbon (see text for details).

## Discussion

**Figure 3:**
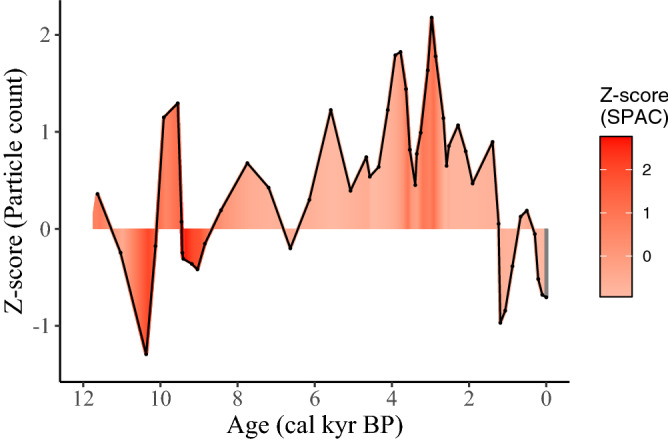
Change in charcoal particle count Z-score over the Holocene with colour mapped according to the Z-score of the mass accumulation rate of SPAC. The most recent sample, representative of a European-induced fire regime is removed from the colour gradient and represented as grey, due to its much higher Z-score (SPAC) of 6.5.

At the beginning of the Holocene, the tropical savanna landscape around Girraween Lagoon was dominated by grasses with relatively little woody vegetation as indicated by *f*_Grass_ ~ 90%. Fires at this time were rare, but intense when they occurred (Fig. 3). This fire regime was in place during a time when the monsoon was relatively suppressed and is consistent with recent analysis that rare, intense, large fires (RIL) are characteristic of dry tropical environments below an apparent precipitation threshold (550 mm yr^−1^)^3^. Although, these authors concluded that Australia was atypical, in that RIL fires could occur in a more mesic tropical environment, we show here that RIL fires were more characteristic of the region when the climate was drier in the early Holocene. Fire intensities abruptly declined as the monsoon increased in intensity, evidenced by lower *δ*D_wax_ values, after ~ 8 cal kyr BP as Girraween lagoon became a larger body of permanent water^12^. This is in agreement with an abrupt increase in precipitation recorded by speleothems just west of our site at this time^18^.

A wetter mid-Holocene is consistent with regionally observed increases in precipitation and temperature, and lower seasonality^19^. In response to this wetter climate, woody vegetation thickened, and fires became more frequent. These fires were of relatively lower intensities, with a higher proportion of woody biomass in the fuel mix. Over the early to mid-Holocene, until roughly 3 cal kyr BP, vegetation cover, vegetation burnt, and fire intensity are all strongly and significantly correlated with the strength of the monsoon as represented by *δ*D_wax_ values (Table 1). Over the wet mid-Holocene interval, fire frequencies increased in tandem with increased woody vegetation, but fire intensities abruptly decreased. Thus, we recognize at least two unique fire regimes over the early to mid-Holocene, RIL fires associated with drier climate and a grassy landscape, and a fire regime of lower intensity, but with more frequent fires associated with a wetter climate and more woody landscape, possibly a Frequent or Intermediate Cool Small (FCS or ICS) pyrome^20^ (Fig. 3).

The onset or strengthening of ENSO variability at ~ 4 cal kyr BP,peaking at ~ 1 cal kyr BP, is well documented^21–24^ and is evidenced in our record by relatively more positive *δ*D_wax_ values with increased temporal variability in Girraween lagoon record. This ENSO-dominated climate was characterized by high inter-seasonal variability and periodic drought across northern Australia^25–27^. A number of responses are apparent in the proxies for fire at Girraween lagoon as ENSO abruptly strengthened. A second increase in fire intensity began at approximately 4 cal kyr BP and peaked at ~ 3 cal kyr BP coeval with a peak in fire frequency. At 3 cal kyr, a sharp and relatively short increase in the proportion of woody biomass burnt is apparent; however, the trend toward woody thickening continues much longer, until ~ 1 cal kyr BP. Thus, a short-lived, fire regime characterized by intense and frequent fires is apparent, beginning just after 4 cal kyr BP and lasting for ~ 1000 yrs. This is consistent with a globally reported increase in fire incidence in the tropics associated either with or just after contemporary El Niño events^28^.

After 3 cal kyr BP, fire intensity abruptly dropped to low values, similar to the mid-Holocene, even as fire frequency remained high. Perhaps most interestingly, is that at this time, the vegetation being burnt transitioned to a fuel mix composed dominantly of understory grasses (*δ*^13^C_SPAC_ values c. − 17‰), even as trees continued to increase their representation on the landscape. Comparison of late Holocene proxies from Girraween Lagoon indicates that there are no longer significant relationships between monsoon (*δ*D_wax_) and vegetation, nor with any attributes of fire regime (Table 1). Moreover, significant relationships between *f*_Grass_ and CHAR, *f*_Grass_ and *δ*^13^C_SPAC_, and MAR_SPAC_ and *δ*^13^C_SPAC_ each changed the direction in correlation slope over the late Holocene, when compared with the early to mid-Holocene. From this we infer, that a new and a very different fire/environmental relationship is evident after 3 cal kyr BP, even though a drier climate became established, similar to that of the early Holocene.

We consider the most parsimonious explanation for the late Holocene decoupling of vegetation and fire from climate to be that human fire management began or intensified, with the imposition of a new and human-mediated fire regime. There is little doubt that the modern indigenous fire regime in tropical Australia is distinct from an unmanaged natural fire regime^2^. The late Holocene fire regime was distinguished by frequent fires of low intensity that primarily burnt the grassy understory. The change to a dominant indigenous fire management in the Late Holocene may have been a response to changing climate variability brought on by droughts associated with ENSO, or may have been due increases in local population bringing more frequent fire management interventions to the landscape near Girraween Lagoon, or both. Indeed, intensification of occupation in the local region is known to have occurred beginning at around 3 kyr BP^29,30^, as well as more broadly in Australia^31^. As sea-levels stabilized from 3 to 2 kyr BP, people might have been encouraged inland to use more woodland sites like those near Girrwaeen^12^. It has also been found that indigenous fire use is more pronounced during periods of climatic variability, presumably to increase landscape productivity^32^.

This study is consistent with Holocene pollen-charcoal research undertaken in the Northern Territory east of Girraween, whereby shifts in vegetation (tree and shrub composition) are linked to a changed fire regime post 3 kyr BP, associated with intensified disturbance as a result of more frequent storm events and human impact^5,33^. There is also evidence from southeast Australia that indigenous fire management intensified beginning at about 3 cal kyr BP^34^ indicating that increased fire management may have been a broader continental phenomenon and that technological and behavioral change accelerated with the onset and intensification of ENSO throughout Australia^31^.

Australia is the most flammable country in the world^20,35^, evident most recently in multiple catastrophic fire seasons^36^. Since European settlement of Australia, and the subsequent replacement of indigenous fire management, fire severity on the continent has increased, as fuel loads increased with fire suppression^7^. Notably, the reintroduction of indigenous fire management practices in northern Australia have led to a reduction in severe fires^8,37^, as well as leading to other ecological benefits^6^. We show that with the intensification of ENSO at 4 cal kyr BP, the ‘natural’ fire regime became severe with both hot and frequent fires. However, shortly after 3 cal kyr BP, the fire regime abruptly changed to one of frequent but mild intensity similar to the characteristics of the mid-Holocene fire regime, but under a new and more variable climate.

We conclude that indigenous fire management became the dominant control on fire regime after ~ 3 cal kyr BP at Girraween Lagoon, an environment typical of northern Australian mesic savannas. Dominant anthropogenic interventions likely resulted in an increase in the frequency and patchiness of fire, limiting fuel accumulation and connectivity respectively, and the targeting of understory grasses. These interventions, in combination, decreased the danger of large, unmanaged, intense fires through the development of a network of heterogenous patches, in turn increasing local floral and faunal biodiversity^6^. Although the resolution of our record does not enable a detailed assessment of the more recently imposed European fire regime, we do note that fire intensity is the highest in the record for the most recent sample (Fig. 3), consistent with other observations of increasing fire intensity accompanying the loss of fine scale, active indigenous fire management in Australia over the last century^5,7,37^.

## Methods

### Lake coring and sediment sampling

Girraween Lagoon is located 12.517° S, 131.081° E just south of Darwin, Australia (Fig. 1). Details of study site, climate, geology, coring and sampling have been previously described^12^, but it is notable that the catchment area is small and our proxies are not overly subject to changing catchment size over time. Briefly, a continuous series of 1 m sediment cores were taken in plastic tubes, using a hydraulic coring rig on a floating platform to a depth of 19.4 m. These core sections were split, described, and sampled in 5–10 cm intervals for the analyses below. Only the Holocene section of the core (approximately the first 5 m) was investigated in this study.

### Radiocarbon analysis and chronology

AMS ^14^C measurements were made as published previously^12,38^ and used for age-model construction. Briefly, samples underwent hydrogen pyrolysis as described below in order to date the SPAC fraction. SPAC samples were combusted to CO_2_ and reduced to a graphite target for measurement at ANSTO using the H_2_/Fe method^39^. A Bayesian approach was used for age modelling. We used the package rbacon^40^ to interpolate calibrated ages determined via the SHCal20^41^ calibration for depth probability distributions of age in R 4.0.0^42^. This age-model is unique from that presented in a previous Girraween pollen record, as described in a subsequent report that focused on the LGM that constructed a revised age-model with new radiocarbon dates added (Supplementary Figure S1).

### *δ*D analysis of *n*-alkanes

Hydrogen isotope analysis of *n*-alkanes in 20 selected sediment samples were undertaken at GFZ Potsdam. Extraction was performed with a dichloromethane (DCM): methanol mixture (9:1 v/v) using an accelerated solvent extraction system kept at 100 °C and 103 bar. Total lipid extracts were separated using 8 mL glass columns filled with ~ 2 g silica gel by solid phase extraction following the manual SPE procedure^43^. Elution proceeded using hexane, Hexane:DCM (1:1 v/v) and DCM as solvents, to recover aliphatic, aromatic, and alcohol/fatty acid fractions, respectively. Compound-specific hydrogen isotope ratios of the aliphatic fraction were measured using a ThermoFisher Delta-V^Plus^ Isotope Ratio Mass Spectrometer coupled to an Agilent 6890 N gas chromatograph at GFZ Potsdam. A standard (‘A4’ obtained from A. Schimmelmann, Indiana University) containing C_16_ to C_30_ *n*-alkanes with known *δ*D values was measured in duplicate at the beginning and the end of each sequence or after six sample injections and used for of *δ*D values normalized to the Vienna Standard Mean Ocean Water (VSMOW) scale. We present *n-*C_27_ and *n-*C_29_ *n*-alkanes as representative of high molecular weight n-alkanes sourced from terrestrial vegetation.

### Stable polycyclic aromatic carbon (SPAC) abundance and carbon isotope analysis

A total of 49 samples for SPAC abundance and carbon isotope composition, representing the Holocene section of the Girraween core are presented in this study. SPAC represents a stable form of pyrogenic carbon whose production in a fire follows a sigmoidal curve with increasing pyrolysis temperature^14,44^. At low temperature, very little SPAC is produced (though blackened char particles are produced), and at mid to higher temperatures 50–100% of total charcoal is measured as SPAC^14,44^. Thus, a greater amount of SPAC is produced with higher intensity fires, whereas low intensity fires produce material counted visually as char, but containing very low SPAC abundance. By contrast, charcoal particles are produced at all temperatures and thus reflects general fire frequency. This proxy is similar to PAH ratios used to determine changes in Indonesian fire regimes over the past 22,000 years^45^. For this study, samples were decarbonated by immersion in 2 M HCl for 3 h and measured for the total organic carbon abundance (TOC). The SPAC component was isolated by hydrogen pyrolysis, now an established method for quantifying pyrogenic carbon^13,14,46^. Approximately 250 mg of the sediment was mixed with a Mo catalyst using an aqueous methanol solution of ammonium dioxydithiomolybdate, sonicated and dried at 60 °C overnight. The sample/catalyst mixture was placed in a reactor, pressurized with H_2_ to 150 bar with a purge gas flow of 5 L min^−1^ and heated at 300 °C min^−1^ to 250 °C and then at 8 °C min^−1^ to 550 °C, held for 5 min. Labile carbon is removed during the hydrogen pyrolysis reaction, and the remaining carbon is composed of a stable form of pyrogenic carbon with greater than seven condensed aromatic rings, or SPAC^14,15,47^. Carbon abundances of Total Organic Carbon (TOC) and SPAC (required for calculation of MAR_SPAC_) and isotope compositions of SPAC were measured via elemental analysis isotope ratio mass spectrometry (EA-IRMS) using a ThermoScientific Flash EA with Smart EA option coupled with a Conflo IV to a Delta V^Plus^ at James Cook University’s Advanced Analytical Centre. Carbon abundances were determined using a TCD (Thermal Conductivity Device). Carbon isotope measurements are reported as per mil (‰) deviations from the VPDB (Vienna Pee Dee Belemnite) reference standard scale for *δ*^13^C values. We used USGS-40 and two internal laboratory reference materials (Taipan, Chitin) within each analytical sequence for 3-point calibrations (normalization) of isotope delta-scale anchored by VPDB. Our internal standards were calibrated using USGS40 and USGS41 international reference materials. SPAC abundances were corrected for possible in situ production of SPAC during the hydrogen pyrolysis reaction, and errors estimated for carbon isotope composition^13^.

### Pollen and charcoal analysis

A total of 37 samples for pollen and charcoal analysis representing the Holocene section of the core are presented in this study. Samples of ~ 2 cm^3^ each were processed by suspension in 10% sodium pyrophosphate followed by sieving over 125 and 7 µm mesh. The sediment fraction retained was treated with 10% KOH, 10% HCl then acetolysis for 10 min prior to undergoing separation by heavy liquid using sodium polytungstate (S.G. 2.0). Samples were immersed in the sodium polytungstate and left for 20 min followed by centrifugation at 2000 rpm (2×). A known amount of Lycopodium spores was added to enable calculation of pollen and charcoal abundances, and the sample mounted with glycerol, sealed with paraffin wax. Both pollen grains and charcoal particles were counted along evenly spaced transects at magnification of ×630 on a Zeiss AxioScope A.1. The average number of pollen grains counted per sample was 300. Any sample which did not meet this count was removed from the data set, maintaining high confidence in data analyses. A few samples were discounted in the data analysis as these had low total pollen counts. Pollen percentages were calculated against a pollen sum consisting of all angiosperms and gymnosperms pollen grains counted (that is excluding pteridophyte and fungal spores) excluding those identified as mangrove/coastal species. Percentages of Poaceae and total arboreal pollen grains were compared in order to determine the fraction of terrestrial pollen that is grass (*f*_Grass_). Charcoal fragments larger than 10 µm were counted along three equally spaced transects of each sample slide and presented as flux (number of particles cm^−3^ yr^−1^). Details, including detailed pollen percentages has been previously published^12^.

### Statistical analysis

Generalized Additive Models (GAMs) were used to identify significant periods of change for each proxy in the Girraween record. GAMs are statistical models that can be used to estimate trends as smooth functions of time^48^. All GAMs were fitted using the package mgcv (Mixed GAM Computation Vehicle)^49^ in R4.0.0^42^. The first derivative of the GAM splines was calculated and considered significant when slopes exceeded confidence intervals (significance set to 0.975). Comparison of proxy data was achieved through the application of Pearson’s correlation coefficient. We investigated correlations among every proxy for the entire time series, early to mid-Holocene (prior to 3000 cal yrs BP), and late Holocene (earlier than ~ 3000 cal yrs BP) noting significance and slope.

## Supplementary Information

Supplementary Information.
