# Supplementary material for: Indigenous impacts on north Australian savanna fire regimes over the Holocene
Source: Sci Rep. 2021 Nov 30;11:23157. doi: 10.1038/s41598-021-02618-z (PMC8632886; doi:10.1038/s41598-021-02618-z)
Supplement: Supplementary file 1 — Supplementary Information. [file 41598_2021_2618_MOESM1_ESM.docx]

Indigenous impacts on north Australian savanna fire regimes over the Holocene

Christopher M. Wurster^1,2*^, Cassandra Rowe^1,2^, Costijn Zwart^1,2^, Dirk Sachse^3^, Vladimir Levchenko^4^, Michael I Bird^1,2^


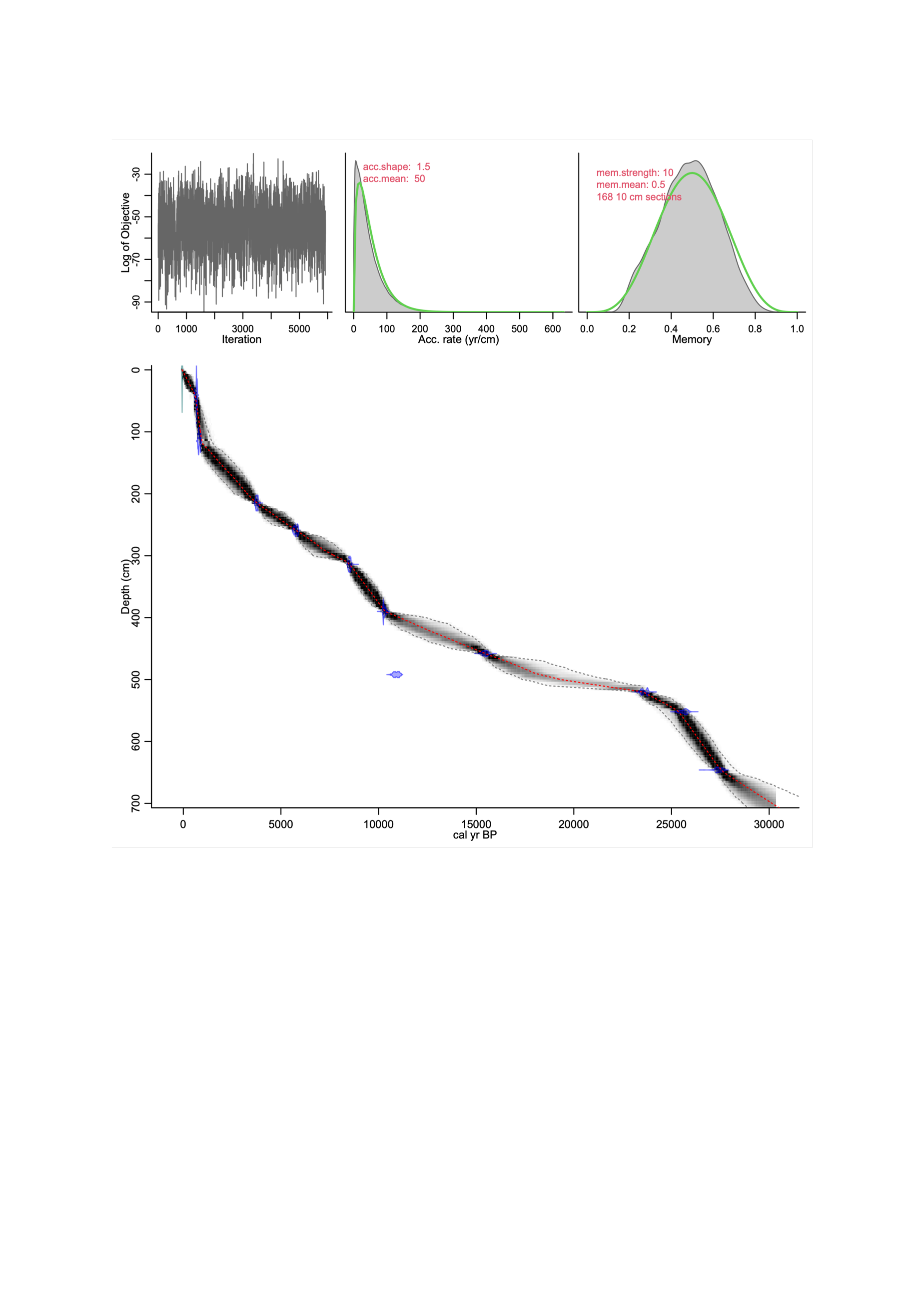


**Figure S1.** Age-depth relationship (using Bacon^1^) output for Girraween Lagoon radiocarbon measurements showing probability distribution of ages as a function of depth combining previously published radiocarbon measurements^2,3^.

**References**

1. Blaauw, M. & Christen, J. A. Flexible paleoclimate age-depth models using an autoregressive gamma process. *Bayesian Anal.* **6**, 457–474 (2011).

2. Rowe, C. *et al.* Holocene savanna dynamics in the seasonal tropics of northern Australia. *Rev. Palaeobot. Palynol.* **267**, 17–31 (2019).

3. Rowe, C. *et al.* Vegetation over the last glacial maximum at Girraween Lagoon, monsoonal northern Australia. *Quat. Res.* 1–14 (2020) doi:10.1017/qua.2020.50.
